# Investigating the Predictive Power of the National Early Warning Score in Predicting Outcomes Among Intensive Care Unit Patients

**DOI:** 10.1155/ccrp/3647049

**Published:** 2026-07-22

**Authors:** Mahmoud Yousefifard, Madeeh Heydarzadeh Jazi, Maryam Esmaeili, Parvaneh Asgari

**Affiliations:** ^1^ Physiology Research Center, Iran University of Medical Sciences, Tehran, Iran, iums.ac.ir; ^2^ Critical Care Department, School of Nursing and Midwifery, Tehran University of Medical Sciences, Tehran, Iran, tums.ac.ir; ^3^ Nursing and Midwifery Care Research Center, Critical Care Department, School of Nursing and Midwifery, Tehran University of Medical Sciences, Tehran, Iran, tums.ac.ir

**Keywords:** death, early warning system, intensive care unit, vital signs

## Abstract

**Aim:**

The aim of this study is to determine the predictive power of the National Early Warning Score in identifying the outcomes of intensive care unit (ICU) patients.

**Methods:**

In this study, 300 patients hospitalized in the ICUs (medical and general) of two teaching hospitals affiliated with Tehran University of Medical Sciences were evaluated and followed up for 30 days between June 2023 and April 2024. The samples were selected by convenience sampling based on inclusion criteria. A checklist containing the components of early warning criteria introduced by Braden, SOFA and APACHE II, as well as outcomes of hospitalization in the ward, was used. The early warning criteria were assessed by the researcher at the time of admission, as well as 6 and 24 h after the admission of patients to ICU. The collected data were analysed by SPSS‐27 and STATA‐14 software using Kruskal–Wallis, Pearson and chi‐square tests, as well as the area under the receiver operating characteristic (ROC) curve of early warning system.

**Results:**

The mean age of patients was 41 years (31–56), and 63.70% of them were male. Comparison of the area under the ROC curve of early warning tool showed that the early warning score in predicting death at the time of admission, 6 h after admission and 24 h after admission was 0.697, 0.712 and 0.87, respectively. Also, this score in predicting adverse outcomes at the time of admission, 6 h after admission and 24 h after admission was 0.700, 0.784 and 0.936, respectively. In other words, the early warning system had a good predictive power in indicating the occurrence of outcomes at all three times (at admission, 6 hours after admission and 24 h after admission), but the best time to predict death and adverse outcomes was estimated to be 24 h after admission.

**Conclusion:**

By evaluating the predictive power of early warning score in identifying the outcomes of patients hospitalized in ICUs, we found that the early warning score at 24 h after admission had a higher predictive power than other times.

## 1. Introduction

Early identification of clinical deterioration remains a cornerstone of critical care practice. Delays in recognizing acute physiological decline are strongly associated with increased risk of cardiac arrest, unplanned intensive care unit (ICU) admission, and mortality [1]. To mitigate these risks, several scoring systems have been developed to provide objective measures of illness severity and guide escalation of care.

The National Early Warning Score (NEWS), and its updated version NEWS2, are widely implemented across the United Kingdom and increasingly adopted internationally as standardized track‐and‐trigger systems [2, 3]. NEWS relies on routinely measured vital signs—respiratory rate, oxygen saturation, systolic blood pressure, pulse rate, temperature, and level of consciousness—and has demonstrated strong predictive value for short‐term adverse outcomes in emergency departments and general wards. Its simplicity and rapid applicability make it attractive for frontline use [4].

However, the utility of NEWS in the ICU population remains less well established. Critically ill patients often present with complex pathophysiology, multiorgan dysfunction, and require advanced monitoring [5]. In this setting, more comprehensive scoring systems such as the Sequential Organ Failure Assessment (SOFA) and the Acute Physiology and Chronic Health Evaluation II (APACHE II) are traditionally used. SOFA quantifies organ dysfunction and has been validated as a predictor of sepsis‐related mortality, while APACHE II integrates a wide range of physiological and laboratory variables collected over 24 h and remains a benchmark for ICU outcome prediction. Both scores, however, are resource intensive and less practical for rapid bedside assessment [6, 7].

Recent comparative studies (2020–2024) have highlighted that while NEWS and NEWS2 retain discriminatory power in predicting ICU mortality and adverse outcomes, their performance relative to SOFA and APACHE II is variable [8–10]. Importantly, most validations of NEWS have been conducted in emergency or ward settings, leaving a gap in evidence regarding its accuracy and clinical utility within the ICU population. Recent studies have demonstrated that the use of NEWS in prehospital emergency settings is effective in predicting mortality and ICU admission within 48 h of hospitalization, supporting its role as a reliable triage tool for acutely ill patients [11–13]. Addressing this gap is critical, if NEWS can reliably predict deterioration in ICU patients, it may serve as a simple adjunct or alternative to complex scoring systems, facilitating timely intervention and resource allocation [14].

Therefore, validating NEWS in the ICU population is valuable not only to determine its prognostic accuracy compared with SOFA and APACHE II but also to clarify its role in critical care pathways [5–7]. Such evidence may inform whether NEWS can be integrated into ICU practice as a rapid screening tool, complementing established severity scores and enhancing patient safety. Most previous studies have examined the predictive power of early warning systems in general and prehospital emergency departments, but few studies have evaluated NEWS specifically in ICU populations. There have also been contradictory findings regarding the impact of early warning systems on patient‐related outcomes and the reliable time for correct prediction. Therefore, the present study aims to determine the predictive power of NEWS in identifying the outcomes of patients hospitalized in the ICU.

## 2. Methods

This is a prospective, single group and descriptive study that was conducted to determine the predictive power of NEWS in identifying the outcomes of patients hospitalized in ICUs in selected hospitals affiliated with Tehran University of Medical Sciences. For this purpose, information of 300 patients were collected between June 2023 and April 2024. The researcher visited the ICUs and after introducing himself to the research samples and providing information about the research objectives and confidentiality of personal information, began to collect the data. Using the data available in the hospital HIS system, ICU documents and patient records, data were collected with use of a questionnaire containing demographic information. In this study, Braden, SOFA and APACHE II criteria were also used at the time of admission and a NEWS assessment checklist was used at the time of admission, as well as 6 and 24 h after admission to the ICU. The method used to assess the early warning criteria was based on a standard protocol that included seven components; respiratory rate, arterial blood oxygen saturation, supplemental oxygen intake, temperature, systolic blood pressure, heart rate and level of consciousness. The patients studied were followed up from the time of admission to ICUs until a maximum of 30 days after admission. The outcomes evaluated based on early warning criteria included death, heart failure (HF) and transfer to the regular ward.

### 2.1. Setting and Samples

Patients aged 18–60 years admitted to the ICU were selected by the convenience sampling method. Pregnant patients, patients with a history of HF, patients requiring palliative care and patients undergoing surgery were not included in the study. Patients who had been admitted to ICU for more than 30 days were excluded from the study.

### 2.2. Data Collection Tools

Demographic information (age, gender, education level, marital status and body mass index) as well as history of underlying disease, medication use, number of hospitalizations, history of substance abuse, diagnosis by the attending physician, medications used in the ward, date of admission, and length of ICU stay were collected. In addition, a checklist containing components of Braden, SOFA, and APACHE II criteria was used at admission. Meanwhile, a NEWS assessment checklist was used at admission, 6 h after admission and 24 h after admission to the ICU. Data were collected by 2 trained nurses in the ICU who were fully familiar with the data collection tools. Before the study, in order to confirm the inter‐rater reliability of the 2 nurses in scoring the NEWS and other tools, a preliminary study was conducted in which both nurses assessed the scores simultaneously for 15 similar patients, obtaining the kappa coefficient of 91%.

### 2.3. Data Analysis

The data were entered into STATA statistical software Version 18. Descriptive statistics (frequency and frequency percentage for qualitative data, mean and standard deviation for quantitative data with normal distribution and median and interquartile range for quantitative data with nonnormal distribution) were used to describe the research units. Chi‐square or Fisher’s exact test was used to compare qualitative and nominal data. Cronbach’s alpha coefficient was calculated to evaluate the internal consistency of the scoring criteria.

The primary outcome was adverse outcome defined as death or HF during hospitalization. The discriminatory performance of the NEWS measured at admission (0 h), 6h and 24 h was evaluated using receiver operating characteristic (ROC) curves. Areas under the ROC curve (AUCs) were reported with 95% confidence intervals (CIs). AUCs were compared using the DeLong test for correlated ROC curves. For NEWS at 24 h, diagnostic performance across a range of cutoff values was summarized using sensitivity and specificity; the optimal cutoff was selected based on the maximum Youden index (sensitivity + specificity − 1). Likelihood ratios (LR+ and LR−) were also reported.

To assess whether NEWS at 24 h independently predicted adverse outcome, multivariable logistic regression models were fitted and results were reported as odds ratios (ORs) with 95% CIs. Two models were specified: Model 1 adjusted for demographic and clinical covariates (age, sex, BMI category, comorbidity history, drug history, smoking, alcohol use and substance use) and Model 2 additionally incorporated severity scores (SOFA and APACHE II) alongside age and sex. A two‐sided *p* value < 0.05 was considered statistically significant.

### 2.4. Ethical and Institutional Approvals

This study was approved by the Ethics Committee of Tehran University of Medical Sciences with the ethics code: IR.TUMS.FNM.REC.1402.057 On May 28, 2023. Throughout the study, the researchers adhered to the principles outlined in the Declaration of Helsinki. Informed consent was obtained from the patients or their legal guardian before entering the study. Confidentiality of patient information was maintained throughout the data collection process.

## 3. Results

### 3.1. Demographic and Clinical Information

In the present study, the data of 300 patients were reviewed. The mean age of patients was 41 (31–56) years, and 63.70% of them were male. Other demographic characteristics of the patients are listed in Table 1. The median of NEWS at admission was 7 with an interquartile range of 5–9 (indicating high risk). The NEWS 6 h after admission was 6, with an interquartile range of 3–8 (indicating moderate risk), and 24 h after admission, the median of NEWS was 5 with an interquartile range of 2‐9es (indicating moderate risk). The APACHE II score of patients was also 9 and their Braden score was 11 (Table 2). Overall, 184 (61.3%) had a favourable outcome, while 116 (38.7%) experienced an adverse outcome, including 97 deaths (32.3%) and 19 cases of HF (6.3%).

**TABLE 1:** Demographic and baseline characteristics of the patients.

Variable	Frequency	Percentage
Sex		
Female	109	36.30
Male	191	63.70
Total	300	100.00
Education		
Illiterate	49	16.33
Primary school	71	23.67
Diploma	84	28.00
University	96	32.00
Total	300	100.00
Marital status		
Single	84	28.00
Married	200	66.67
Spouse deceased	6	2.00
Divorced	10	3.33
Total	300	100.00
Body mass index (BMI)		
Normal (18.5–24.9)	185	61.67
Overweight (25–29.9)	101	33.67
Obesity ≥ 30	14	4.67
Total	300	100.00
History of underlying disease		
Diabetes	14	4.67
High blood pressure	4	1.33
Pulmonary disease	6	2.00
Cardiac disease	1	0.33
Two or more diseases	107	35.67
Other diseases	42	14.00
No disease	126	42.00
Total	300	100.00
Drug use history		
Cardiac	4	1.33
Pulmonary	6	2.00
Neurological	13	4.33
Diabetes	8	2.67
Two or more drugs	113	37.67
Other	16	5.33
No drug history	140	46.66
Total	300	100.00
Drugs received in the unit		
Inotropic	1	0.33
Two or more drugs	295	98.33
Other	4	1.33
Total	300	100.00

**TABLE 2:** Median and interquartile range of SOFA, APACHE II and Braden tools at admission, and early warning score at admission, 6h and 24 h after admission.

Variable	Median	Interquartile range
EWS0	7	5–9
EWS6	6	3–8
EWS24	5	2–9
SOFA	8	6–10
APACHE II	9	6–13
BRADEN	11	9–15.5

### 3.2. National Early Warning Score in Predicting Death and HF

**FIGURE 1:**
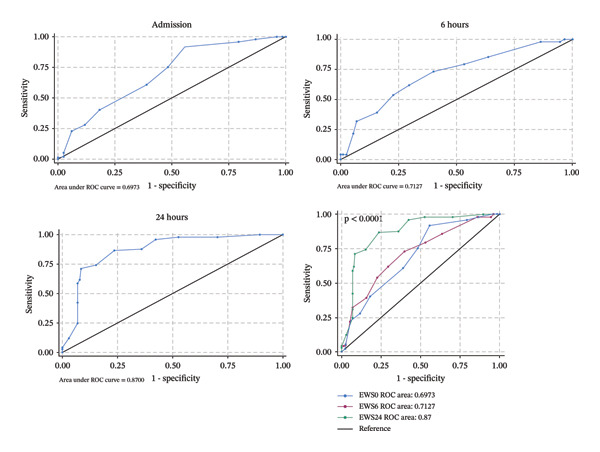
NEWS prediction of death at 0, 6 and 24 h after admission.

**FIGURE 2:**
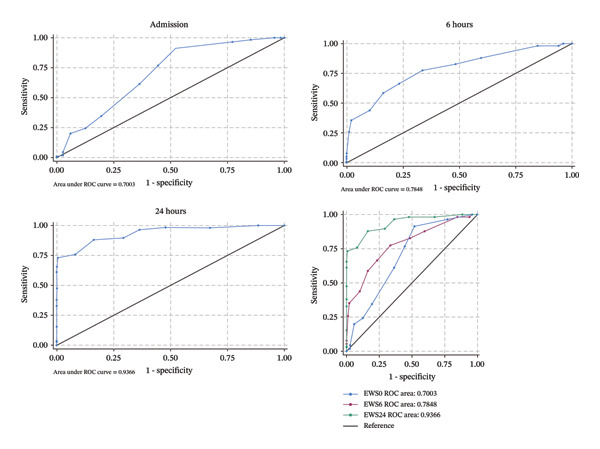
NEWS prediction of adverse outcome (death and heart failure) at time 0, 6 and 24 h after admission.

The area under the curves reported for NEWS in predicting death at time 0, 6 and 24 h after admission with a 95% CI was calculated as 0.697, 0.712 and 0.87, respectively (Figure 1). The area under the curve for NEWS in predicting adverse outcome at time 0, 6 and 24 h after admission was also determined to be 0.700, 0.784 and 0.936, respectively (Figure 2). The findings showed that the NEWS did not differ in predicting patient outcomes at the time of admission and 6 hours after admission, but it did differ at 24 h after admission, highlighting that the best time to predict hospitalization outcomes by this tool is 24 h after admission. Also, the predictive value of aforementioned scale at different cutoff points showed that the highest sensitivity and specificity at 24 h after admission belonged to the score of 4 (Table 3).

**TABLE 3:** Predictive value of NEWS 24 h after admission at different cutoff points in relation to adverse outcome.

Cutoff point	Sensitivity	Specificity	Positive likelihood ratio (LR+)	Negative likelihood ratio (LR−)
0 ≤	100.0	0.00	1.00	—
1 ≤	100.0	11.41	1.12	0.00
2 ≤	98.28	32.61	1.45	0.05
3 ≤	28.98	52.17	2.05	0.03
4 ≤	69.55	63.59	2.65	0.05
5 ≤	89.66	70.65	3.05	0.14
6 ≤	87.93	83.70	5.39	0.14
7 ≤	75.86	91.85	9.30	0.26
8 ≤	73.28	99.46	134.82	0.26
9 ≤	65.52	100.00	—^∗^	0.34
10 ≤	61.21	100.00	—	0.38
11 ≤	47.41	100.00	—	0.52
12 ≤	37.93	100.00	—	0.62
13 ≤	32.76	100.00	—	0.67
14 ≤	15.52	100.00	—	0.84
15 ≤	3.45	100.00	—	0.96
16 ≤	2.59	100.00	—	0.97
17 ≤	0.00	100.00	—	1.0

^∗^These values are not calculable due to the absence of cases in that specific range.

### 3.3. Comparison of the AUCs for NEWS in Predicting Adverse Outcomes at Different Times

**FIGURE 3:**
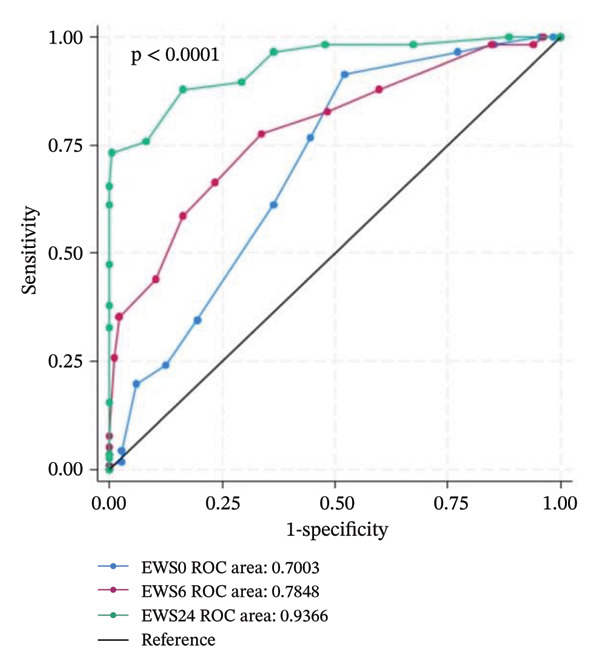
Comparison of the area under the ROC curves of NEWS in predicting favourable and unfavourable outcomes at different times.

Figure 3 shows the comparison of the area under the curve for NEWS at the time of admission, 6 h after admission and 24 h after admission. As seen in the figure, the area under the curve for NEWS at the time of admission was 0.700, 6 h after admission was 0.784 and 24 h after admission was 0.936. The comparison showed no difference between the area under the curve at the time of admission and 6 h after admission, but the area under the curve at 24 h after admission was significantly different from other times (Figure 3). Therefore, it can be said that the best time to predict favourable and adverse outcomes of patients in the ICU is 24 h after their admission.

### 3.4. Cutoff Performance for NEWS at 24 h

The optimal cutoff (Youden index) for NEWS at 24 h was ≥ 8, corresponding to a sensitivity of 73.28% and specificity of 99.46%, with positive LR 134.82 and negative LR 0.26. For settings prioritizing higher sensitivity for screening preference, a lower cutoff of ≥ 4 yielded sensitivity of 96.55% and specificity of 63.59% (Positive LR = 2.65 and Negative LR = 0.05) (Table 3).

### 3.5. Comparison With NEWS at 24 h, SOFA and APACHE II

For adverse outcome, the AUC of SOFA was 0.708 (95% CI: 0.648–0.767) and the AUC of APACHE II was 0.716 (95% CI: 0.660–0.773). In comparison, NEWS at 24 h achieved an AUC of 0.937 (95% CI: 0.908–0.965), indicating substantially better discrimination.

### 3.6. Multivariable Logistic Regression

In multivariable analysis (Model 1: demographic/clinical covariates), NEWS at 24 h remained an independent predictor of adverse outcome (OR 2.28, 95% CI: 1.85–2.81; *p* < 0.001) (Table 4). In this model, current smoking status was inversely associated with adverse outcome (OR 0.05, 95% CI: 0.00–0.54; *p* = 0.014), while other covariates did not reach statistical significance. The model showed excellent discrimination (AUC = 0.960). In the severity‐adjusted model (Model 2: NEWS 24 h + SOFA + APACHE II + age + sex), NEWS at 24 h remained strongly associated with adverse outcome (OR 2.25, 95% CI: 1.85–2.73; *p* < 0.001) (Table 5). Male sex was also significantly associated with adverse outcome (OR 2.46, 95% CI: 1.04–5.83; *p* = 0.041), whereas SOFA (OR 0.87, 95% CI: 0.75–1.02; *p* = 0.092) and APACHE II (OR 1.00, 95% CI: 0.91–1.11; *p* = 0.950) were not significant after adjustment. Model 2 maintained strong discrimination (AUC = 0.946).

**TABLE 4:** Multivariable logistic regression (clinical covariates) for predicting adverse outcome (death or heart failure).

Predictor	OR (95% CI)	*p* value
NEWS at 24 h (per 1‐point increase)	2.28 (1.85 to 2.81)	< 0.001
Age (years, per 1‐year increase)	1.00 (0.96 to 1.04)	0.873
Male sex (vs. female)	2.19 (0.84 to 5.73)	0.109
Any comorbidity (yes vs. no)	0.25 (0.03 to 2.16)	0.209
Drug use history (yes vs. no)	4.82 (0.57 to 40.59)	0.148
Smoking (yes vs. no)	0.05 (< 0.01 to 0.54)	0.014
Alcohol use (yes vs. no)	3.75 (0.91 to 15.41)	0.066
Substance use (yes vs. no)	2.05 (0.19 to 21.66)	0.550
Overweight (BMI 25–29.9 vs. normal)	2.71 (0.95 to 7.78)	0.063
Obesity (BMI ≥ 30 vs. normal)	0.23 (0.01 to 9.34)	0.441

Abbreviations: CI, confidence interval; OR, odds ratio.

**TABLE 5:** Multivariable logistic regression including SOFA and APACHE II for predicting adverse outcome (death or heart failure).

Predictor	OR (95% CI)	*p* value
NEWS at 24 h (per 1‐point increase)	2.25 (1.85 to 2.73)	< 0.001
SOFA score (per 1‐point increase)	0.87 (0.75 to 1.02)	0.092
APACHE II score (per 1‐point increase)	1.00 (0.91 to 1.11)	0.950
Age (years, per 1‐year increase)	1.02 (0.99 to 1.06)	0.174
Male sex (vs. female)	2.46 (1.04 to 5.83)	0.041

## 4. Discussion

This study demonstrates that the prognostic performance of the NEWS in critically ill patients is strongly time dependent, with substantially improved discrimination for both mortality and adverse outcomes when assessed 24 h after ICU admission, compared with measurements obtained at admission and 6 h thereafter. Importantly, NEWS at 24 h outperformed established severity scores, including SOFA and APACHE II, and remained a robust independent predictor of adverse outcomes after multivariable adjustment. These findings support the growing recognition that dynamic physiological assessment provides greater prognostic value than single time‐point severity scoring in critical care settings.

At ICU admission and 6 h after admission, NEWS demonstrated only moderate discriminatory ability. This finding is consistent with recent large‐scale evaluations of early warning systems, which report modest prognostic accuracy when scores are calculated early in the disease course or during initial resuscitation phases [15, 16]. Early physiological measurements in critically ill patients are influenced by multiple transient factors, including prehospital care, sedation, vasopressor use, and early therapeutic interventions, which may obscure the underlying severity and trajectory of illness. In contrast, NEWS measured at 24 h after ICU admission achieved excellent discrimination for both mortality and adverse outcomes. This substantial improvement likely reflects the stabilization period during which patients’ physiological responses to treatment become evident. By this time, persistent derangements in vital signs may represent true disease progression or treatment failure rather than reversible acute instability. Recent systematic reviews and observational studies emphasize that serial and delayed NEWS assessments outperform admission values, reinforcing the importance of repeated measurement over time [17, 18].

The comparative analysis of AUCs across time points demonstrated that NEWS assessed at 24 h was significantly superior to NEWS measured at admission or 6 h, while no meaningful difference was observed between the two earlier measurements. These findings suggest that admission NEWS, although valuable for early detection of deterioration, may be inadequate as a standalone prognostic indicator in the ICU. Instead, reassessment at 24 h appears to provide an optimal window for outcome prediction, consistent with contemporary evidence supporting dynamic risk stratification models in critical illness [17]. This observation aligns with the broader critical care paradigm in which established severity scores, such as APACHE II, rely on the worst values obtained during the first 24 h to enhance prognostic accuracy. However, unlike APACHE II, NEWS offers the advantage of simplicity, bedside availability and repeatability without reliance on laboratory data.

The analysis of NEWS cutoff values at 24 h highlights its flexibility for different clinical objectives. A higher threshold (NEWS ≥ 8) demonstrated excellent specificity and a very high positive likelihood ratio, making it suitable for identifying patients at extremely high risk of adverse outcomes who may benefit from urgent escalation of care or closer monitoring. Similar findings have been reported in contemporary studies evaluating NEWS and NEWS2 in acute and critical care populations [17, 19]. Conversely, a lower cutoff (NEWS ≥ 4) provided excellent sensitivity, supporting its use as a screening threshold when the goal is early identification and minimization of missed deterioration. This dual‐threshold approach is consistent with recent recommendations advocating context‐dependent NEWS interpretation, allowing clinicians to balance sensitivity and specificity according to clinical priorities [17, 20].

A key contribution of this study is the demonstration that NEWS at 24 h outperformed SOFA and APACHE II in predicting adverse outcomes. While SOFA and APACHE II remain central to ICU prognostication, they are more complex, require laboratory data and may be less responsive to short‐term physiological trends. Recent comparative studies indicate that simplified early warning systems, when applied dynamically, can achieve comparable or superior discrimination to traditional ICU severity scores [15, 21, 22].

The finding that SOFA and APACHE II were not independently associated with adverse outcomes after adjustment, whereas NEWS at 24 h remained strongly predictive, suggests that NEWS captures clinically meaningful physiological instability not fully accounted for by organ‐failure–based indices. These results support the complementary role of NEWS alongside established severity scores, particularly for ongoing monitoring and real‐time decision‐making.

In multivariable logistic regression analyses, NEWS at 24 h remained a strong independent predictor of adverse outcomes across models, including those adjusted for demographic variables and severity scores. The excellent discriminatory performance of these models further underscores the robustness of NEWS as a prognostic indicator.

The inverse association observed between current smoking status and adverse outcomes should be interpreted cautiously. Similar paradoxical associations have been reported in recent observational studies and are thought to reflect residual confounding, selection bias or collider effects rather than a true protective mechanism [15, 23]. Additionally, male sex was independently associated with adverse outcomes, consistent with recent evidence describing sex‐based differences in critical illness outcomes, potentially related to biological or treatment‐related factors.

## 5. Strengths and Limitations

This study is strengthened by its evaluation of NEWS across multiple time points, direct comparison with established ICU severity scores and use of multivariable models to account for potential confounders. However, several limitations should be noted. The single‐center design may limit generalizability, and residual confounding cannot be excluded despite adjustment. Additionally, the analysis focused on discrete time points rather than continuous NEWS trajectories, which may provide further prognostic insight.

## 6. Implication and Recommendations for Practice

In ICUs, the highest level of care and treatment is provided to critically ill patients with life‐threatening conditions, and early detection of abnormal vital signs is important to prevent complications. Therefore, the existence of a reliable scale in assessing patients can reduce adverse events. With the results obtained in this study, nursing managers and head nurses of ICUs will be able to introduce NEWS as a way of detecting abnormal vital signs and improving patient care conditions. It is suggested that the use of NEWS be investigated in identifying long‐term clinical outcomes of patients.

## 7. Conclusions

The findings of present study showed that NEWS is effective in predicting adverse outcomes (death and HF), and the discharge and transfer of patients from and to ICU. Therefore, nurses can use this tool for early detection of adverse outcomes in ICU patients.

## Funding

No funding was received for this research.

## Ethics Statement

This research project has been approved by a research ethics committee of the Tehran University of Medical Sciences (TUMS) with the ethics code: IR.TUMS.FNM.REC.1402.057 in 2023.

## Consent

All participants or their legal guardians gave informed consent for the research, and their anonymity was preserved.

## Conflicts of Interest

The authors declare no conflicts of interest.

## Data Availability

The data that support the findings of this study are available on request from the corresponding author. The data are not publicly available due to privacy or ethical restrictions.
